# Orientation of Steel Fibers in Magnetically Driven Concrete and Mortar

**DOI:** 10.3390/ma11010170

**Published:** 2018-01-22

**Authors:** Wen Xue, Ju Chen, Fang Xie, Bing Feng

**Affiliations:** 1 School of Civil Engineering and Architecture, Zhejiang University of Science and Technology, Hangzhou 310023, China; xuewen@zzust.edu.cn; 2 Department of Civil Engineering, Zhejiang University, Hangzhou 310058, China; 3 Faculty of Mechanical Engineering & Mechanics, Ningbo University, Ningbo 315211, China; Xiefangyp@163.com; 4 Shaoxing Electric Power Bureau, Shaoxing 312000, China; zepeifb@163.com

**Keywords:** concrete, magnetic force, mortar, orientation, steel fiber

## Abstract

The orientation of steel fibers in magnetically driven concrete and magnetically driven mortar was experimentally studied in this paper using a magnetic method. In the magnetically driven concrete, a steel slag was used to replace the coarse aggregate. In the magnetically driven mortar, steel slag and iron sand were used to replace the fine aggregate. A device was established to provide the magnetic force. The magnetic force was used to rotate the steel fibers. In addition, the magnetic force was also used to vibrate the concrete and mortar. The effect of magnetic force on the orientation of steel fibers was examined by comparing the direction of fibers before and after vibration. The effect of magnetically driven concrete and mortar on the orientation of steel fibers was also examined by comparing specimens to normal concrete and mortar. It is shown that the fibers could rotate about 90° in magnetically driven concrete. It is also shown that the number of fibers rotated in magnetically driven mortar was much more than in mortar vibrated using a shaking table. A splitting test was performed on concrete specimens to investigate the effect of fiber orientation. In addition, a flexural test was also performed on mortar test specimens. It is shown that the orientation of the steel fibers in magnetically driven concrete and mortar affects the strength of the concrete and mortar specimens.

## 1. Introduction

There has been many research studies about steel fiber reinforced concrete (SFRC) in the last four decades [1]. Studies on the orientation of steel fibers to improve the behavior of SFRC has been carried out recently. It was shown that fiber orientation has an effect on the tensile behavior of SFRC. The ultimate strength and post-peak stresses increase with a more favorable orientation of the steel fibers [2,3]. It was also shown that fiber orientation is able to significantly influence the tensile and bending behaviors of fiber reinforced concrete (FRC) by increasing the fiber efficiency to bridge cracks [4,5,6,7,8]. Plagué et al. [9] investigated the influence of fiber orientation on water permeability of FRC structures. Test results indicate that the tensile strength, first crack load, and ultimate load of tie-specimens decrease when the fiber orientation becomes less favorable. A favorable orientation is when fibers are best aligned with the direction of tensile load. The effect of fiber orientation on water permeability is even more significant than on mechanical behavior, as presented by Plagué et al. [9], which means that fiber orientation has an effect on material properties of concrete other than strength. In previous research, fiber orientation was achieved either by adopting different casting methods [10,11,12,13,14] or by extracting specimens from large specimens oriented in different directions with respect to the flow of the material while casting [4,6,15,16].

Abrishambaf et al. [17] used an electromagnetic device to orientate the steel fibers when casting specimens. It was shown that a wide range of fiber orientation profiles could be achieved by using the electromagnetic device. The electromagnetic device is effective in orienting fibers in the direction aligned to the applied magnetic field. However, the wall effects are obvious for ultra-high performance fiber reinforced cementitious composites having a narrow width. The steel fibers close to the surface of the mold cannot rotate freely when subjected to the magnetic field. In this case, aggregates in the concrete will also prevent the steel fibers from rotating freely. This problem may be solved by using magnetically driven concrete (MDC) [18] and magnetically driven mortar (MDM). Since the aggregates in MDC and MDM move under the magnetic force, the steel fibers may be able to rotate freely when subjected to the magnetic field. In addition, movement of the aggregates may also be able to change the direction of the steel fibers. The effect of magnetically driven concrete and mortar on the orientation of steel fibers was explored in this work. Concrete with orientated steel fibers can be applied in the tension zone of beams and slabs. Experimental investigation on slabs with steel reinforcing bars will be studied in the next test program.

## 2. Steel Fiber Orientation in Magnetically Driven Concrete

### 2.1. Experimental Investigation

In total, four series of concrete specimens were tested, namely series MDC-M (steel slag with magnetic orientation), MDC-S (steel slag with shaking table vibration), NC-M (normal concrete with magnetic orientation), and NC-S (normal concrete with shaking table vibration). Each series had twelve specimens. Six specimens were tested at 7 days while another six specimens were tested at 28 days. The content of the concrete is presented in Table 1. The design grade of the concrete was C30. The particle size distribution of the coarse steel slag was obtained by a sieving test and is shown in Table 2. The maximum size of the river stone used was 35 mm.

**Figure 1:**
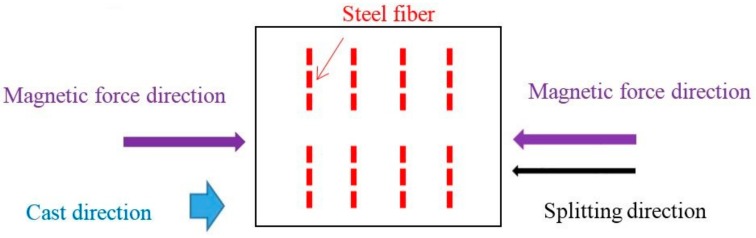
Test setup of fiber orientation in magnetically driven concrete (MDC).

**Figure 2:**
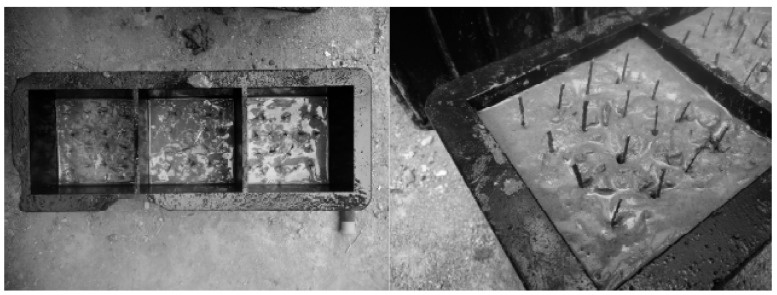
Arrangement of the steel fibers in the concrete.

Steel fibers having a nominal length of 31.0 mm and nominal diameter of 0.689 mm were used. The elastic modulus and tensile strength obtained from the manufacturer were 210 GPa and 810 MPa, respectively. In total, two layers of 32 steel fibers were placed in each 100 × 100 mm cubic concrete specimen. Concrete was cast in the mold to a height of 60 mm and then the first layer of 4 × 4 = 16 steel fibers were placed into the concrete. The height of 60 mm was to leave enough spacing between the bottom plate of the mold and the bottom end of the steel fiber. Since there was no cover plate for the mold, the height of the second layer of concrete was 40 mm. The direction of the steel fibers was vertical to the direction of the magnetic force, as shown in Figure 1. The rest of the concrete was cast into the mold to a height of 100 mm. Another layer of 4 × 4 = 16 steel fibers was placed into the concrete as shown in Figure 2.

**Figure 3:**
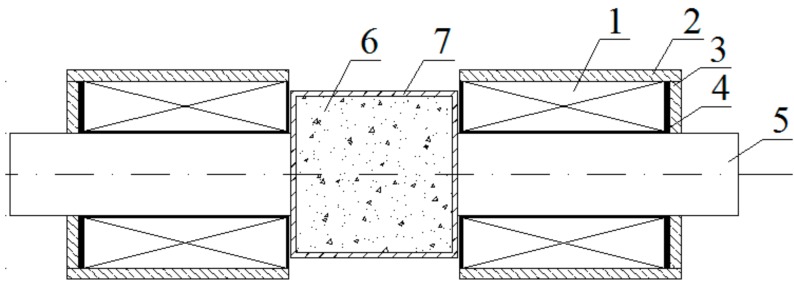
Magnetic vibration device. 1, Coil; 2, Shell; 3, Steel plate; 4, Coil frame; 5, Iron core; 6, Concrete; 7, Mold.

The magnetic field was applied by a device developed by Chen et al. [18] as shown in Figure 3. The direction of the magnetic force was changed by switching the direction of the current at a frequency of 5 s. Each test specimen of series MDC-M was vibrated for about 3 min based on the test results from Chen et al. [18]. Test specimens of series NC-M were vibrated on the shaking table for about 3 min and then the magnetic force was applied to rotate the steel fibers.

**Figure 4:**
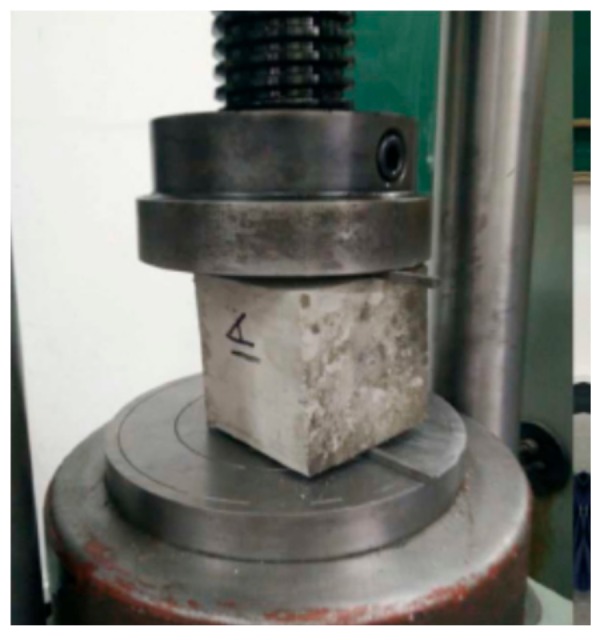
Concrete splitting test.

All concrete cubic specimens were cured in the standard concrete curing room at a temperature of 20 °C and humidity of 90% for 7 and 28 days, respectively. Splitting tests were performed on cubic concrete specimens to obtain the tensile strengths, as shown in Figure 4. The concrete specimens were cured and tested according to the Chinese Standard [19].

### 2.2. Test Results

**Figure 5:**
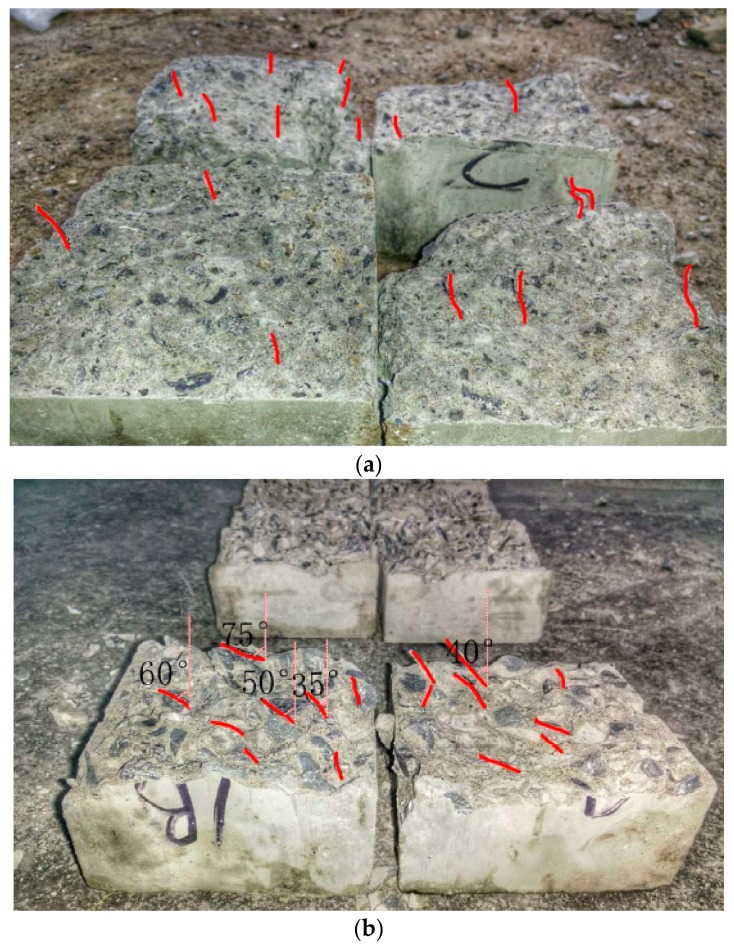

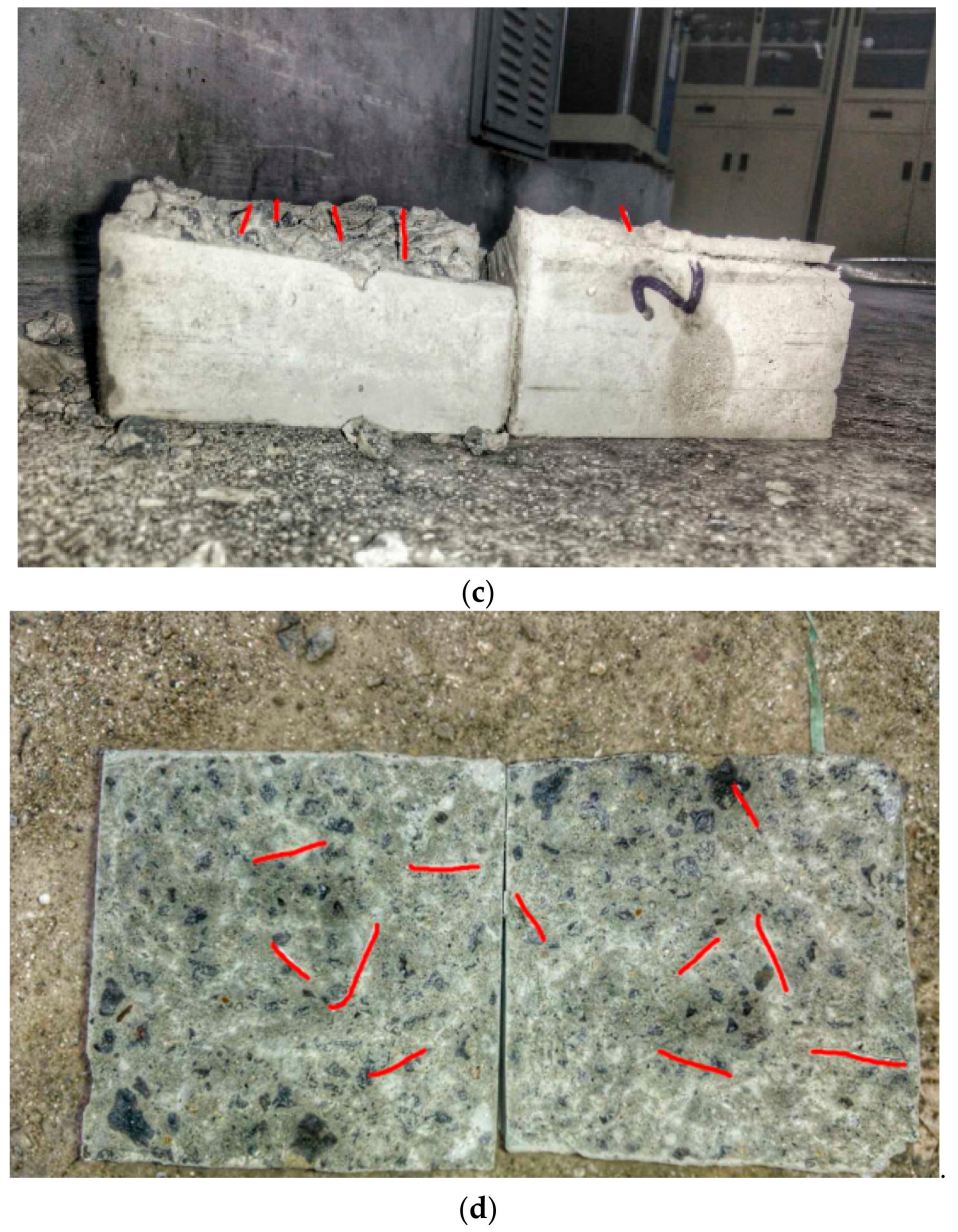
Fiber orientation in different concrete specimens: (**a**) NC-S1; (**b**) NC-M1; (**c**) MDC-S1; (**d**) MDC-M1.

The direction of the steel fibers for specimens NC-S1, NC-M1, MDC-S1, and MDC-M1 are shown in Figure 5a–d, respectively. The direction of the steel fibers of specimen NC-S1 and MDC-S1 remained vertical to the splitting direction, which means that the shaking table had almost no effect on the orientation of the steel fibers. The angles between the steel fibers and the concrete cast direction ranged from 35° to 75° (average value of 45°) for specimen NC-M1. The angles indicate that the magnetic force applied had an effect on the orientation of the steel fibers in normal concrete. For specimen MDC-M1, the steel fibers changed almost 90° after orientation by the magnetic force. A comparison of the four test specimens indicates that the orientation on the steel fibers was most effective in specimen MDC-M1. It may be explained that the movement of the coarse aggregate under the magnetic force was helpful for the steel fiber’s orientation.

The tensile strength obtained from the splitting test could be calculated using Equation (1):

*f*_ts_ = 0.85 × 2*F*/π*A* (1)

where, *f*_ts_ is the tensile strength of the concrete obtained from the splitting test; *F* is the ultimate load obtained from the splitting test; *A* is the cross-section area of the concrete specimen, and 0.85 is the coefficient for 100-mm cubic specimens. Test results of *F* and *f*_ts_ obtained at 7 and 28 days are presented in Table 3, Table 4, Table 5 and Table 6.

Test results obtained from the splitting tests are presented in Table 3, Table 4, Table 5 and Table 6 for specimen series NC-S, NC-M, MDC-S, and MDC-M, respectively. It is shown that the tensile strengths of the specimen series NC-S at both 7 and 28 days were higher than those of the specimen series NC-M by 3.5% and 4.4%, respectively. It is also shown that the tensile strengths of the specimen series MDC-S at both 7 and 28 days were higher than those of specimen series MDC-M by 2.9% and 5.8%, respectively. The enhancement in tensile strength may have been caused by the favorable directions of the steel fibers as shown in Figure 5. Fibers are the best aligned with the direction of tensile load having the maximum contribution in tensile resistance. The angle was measured using a goniometer (Acrobeam, Xi’an, China).

## 3. Steel Fiber Orientation in Magnetically Driven Mortar

### 3.1. Experimental Investigation

**Figure 6:**
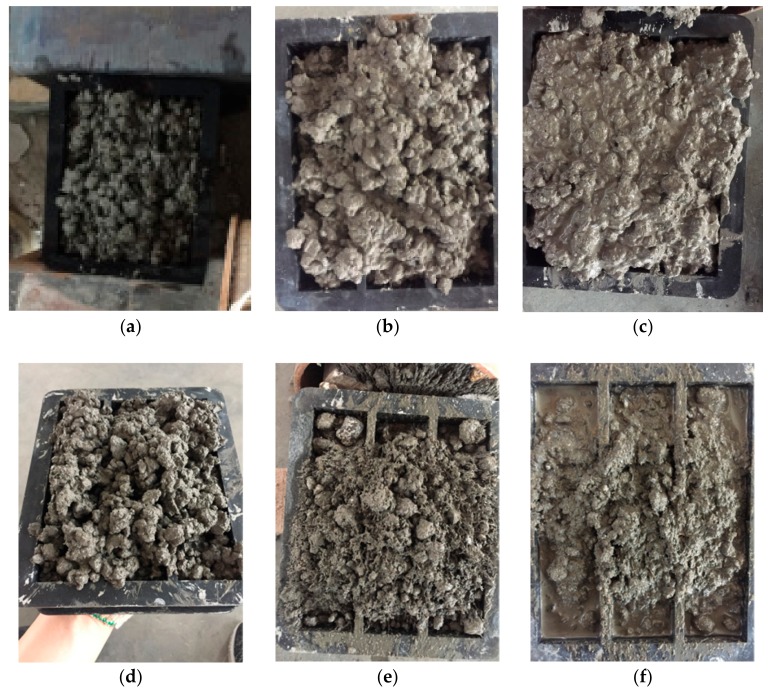
Effect of magnetic vibration for different magnetically driven mortar (MDM) series specimens: (**a**) Series A; (**b**) Series B; (**c**) Series C; (**d**) Series D; (**e**) Series E; (**f**) Series F.

The steel fibers used in the mortar specimens had a nominal length of 10 mm and nominal diameter of 0.02 mm. The elastic modulus and tensile strength obtained from the manufacturer were 205 GPa and 1245 MPa, respectively. A steel slag having a diameter less than 5 mm was used. However, the magnetic force applied to the steel slag was not large enough to vibrate the mortar. Therefore, iron sand was also used in the magnetically driven mortar. Six kinds of mortar were first tried in order to choose the most suitable magnetically driven mortar, as shown in Table 7. The effective amount of sand was investigated in series A, B, C, and D. Series C was suitable as shown in Figure 6c. However, the magnetic force was not enough to vibrate the mortar. Therefore, iron sand was added in series E and F. Test results indicate that the amount of iron sand should be controlled since the adhesive force between iron sand and cement paste is relatively small, as shown in Figure 6e. Finally, series F was used in the fiber orientation test. The iron sand may have corrosion problems on the steel fibers; this should be considered in real applications.

**Figure 7:**
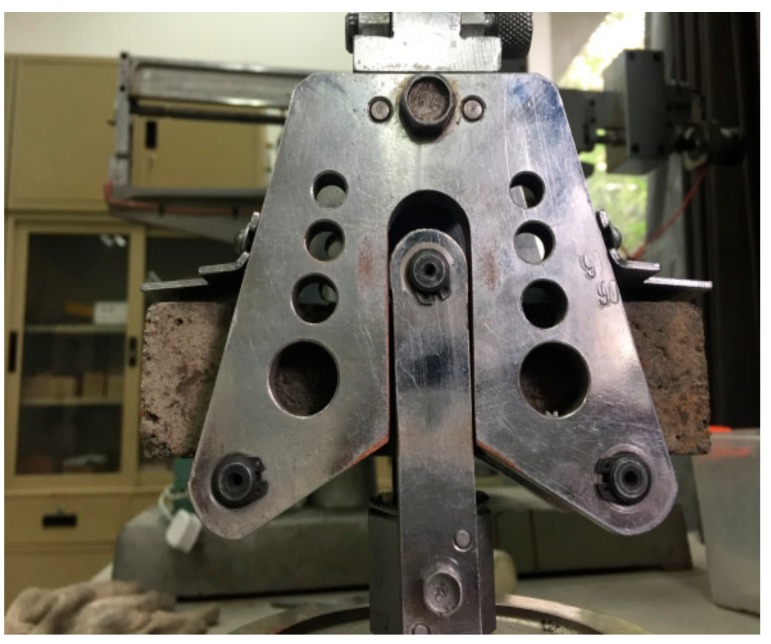
Flexural loading test of specimen.

In total, two groups of ten series of test specimens were fabricated, as shown in Table 8. Group A included five series of specimens with fiber volume fractions (*V*_f_) of 0%, 0.5%, 1.0%, 1.5%, and 2.0% (volume), respectively. All test specimens in group A were vibrated by the magnetic method while all test specimens in group B were vibrated by a shaking table. Group B also contained five series of specimens with *V*_f_ of 0%, 0.5%, 1.0%, 1.5%, and 2.0%, respectively. All test specimens were cured for 7 days before the flexural loading test (shown in Figure 7). The size of all test specimens was 40 × 40 × 160 mm.

### 3.2. Test Results

**Figure 8:**
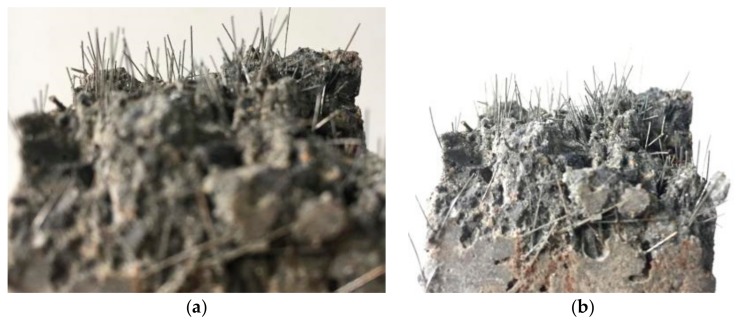
Different orientation of the steel fibers: (**a**) Magnetic method vibration; (**b**) Shaking table vibration.

There are many steel fibers in one mortar specimen, as shown in Figure 8. It is difficult to measure the accurate direction of each steel fiber. The direction of the steel fibers for the specimens was evaluated by the angles between the steel fibers and the magnetic force. The angles were divided into four zones, namely 0°–15°, 15°–45°, 45°–75°, and 75°–90°. The number of steel fibers belonging to each angle zone was counted. The numbers counted for each series of test specimens are presented in Table 9. Steel fibers having angles of 0°–15° are most favorable since these fibers are the best aligned with the direction of the tensile load. It is shown that the numbers in the 0°–15° test specimens vibrated by the magnetic method was much larger than those of the test specimens vibrated by a shaking table. It is shown that the magnetic force could effectively orientate the steel fibers in magnetically driven mortar. There were some voids on the surface of the specimens; however, there were no such voids inside the specimens.

In order to quantitatively evaluate the orientation of the steel fibers, an orientation coefficient ω is proposed as shown in Equation (2):

(2) $$\begin{array}{l} \omega =\frac{\sum_{1}^{n}l\times \cos\theta_{i}}{n\times l}=\frac{1}{n}\sum_{1}^{n}\cos\theta_{i} \end{array}$$

where *l* is the length of the steel fiber; *θ*_i_ is the angle between the steel fiber and the magnetic force direction; *n* is the number of steel fibers.

**Figure 9:**
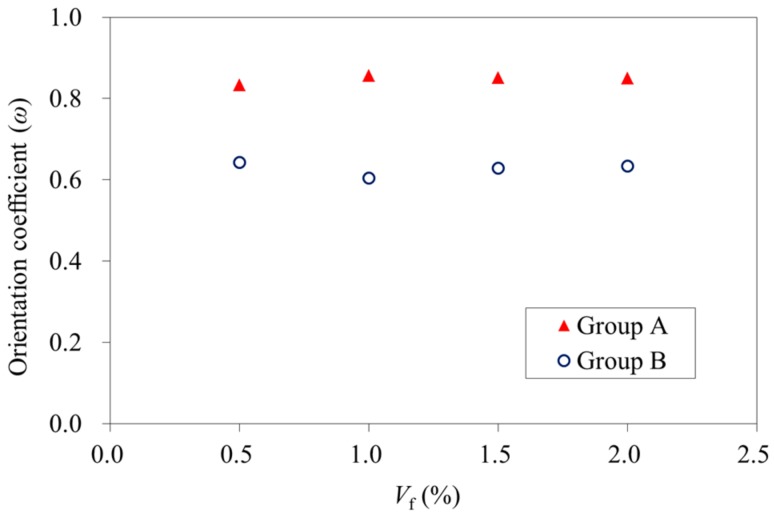
Fiber orientation coefficients of specimens having different fiber volume fractions.

The orientation coefficient ω of each series of test specimens could be calculated using Equation (2) based on the test results in Table 9 and is presented in Figure 9. It is shown that the number of steel fibers had no significant effect on the orientation coefficients. The average values of the orientation coefficients for specimen groups A and B were 0.847 and 0.628, respectively. It is shown that the magnetic force was able to orientate the steel fibers in magnetically driven concrete.

**Figure 10:**
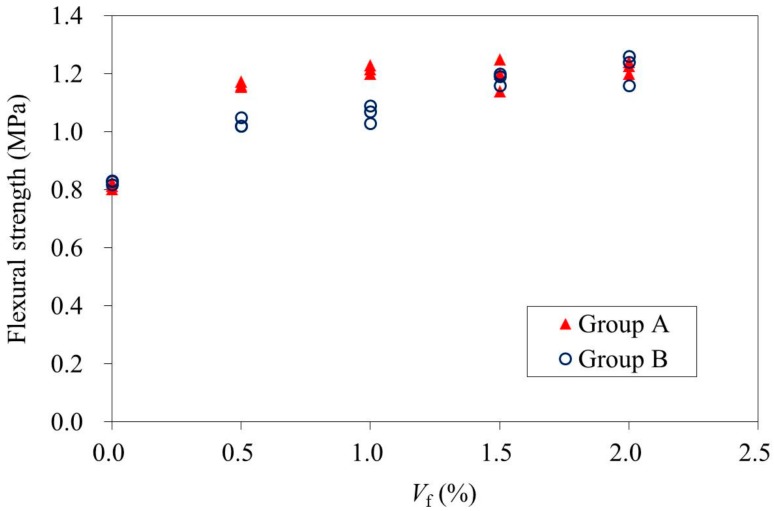
Flexural strengths of specimens having different fiber volume fractions.

The test results obtained from the flexural loading test are shown in Figure 10. Test specimen series having *V*_f_ of 0.5% and 1.0% exhibited obvious differences between groups A and B. As fiber orientation becomes less favorable, the flexural strength characterizing the specimens decreased to 11.5% and 12.1% for the specimen series having a *V*_f_ of 0.5% and 1.0%, respectively. However, there was almost no difference in flexural strength between groups A and B for the specimen series having a *V*_f_ of 1.5% or 2.0%. It is probably related to an insignificant contribution from the fiber orientation since the number of fibers with favorable orientation was enough. It is also shown in Figure 8 that the steel fibers were pulled out from the mortar. If inclined hooked steel fibers are used, an enhancement of the flexural strength may be increased. It needs further investigation. The variation in concrete strength between the specimens may be reflected by the difference among the same specimens. In this case, the difference caused by fiber orientation could be identified, as shown in Figure 10.

## 4. Conclusions

The orientation of the steel fibers in MDC and MDM was investigated in this study. It was shown that the magnetic force was able to force the steel fibers to rotate in normal concrete and MDC. The effect of orientation in MDC was much better than in normal concrete. The fibers in MDC were almost aligned with the direction of the magnetic force after magnetic vibration. As fiber orientation became less favorable in the concrete, the splitting tensile strength decreased.

The best mixture of MDM was obtained from a series of six test specimens. Iron sand was necessary to vibrate the mortar specimen. Magnetic force was able to effectively rotate the steel fibers in MDM. More steel fibers with favorable orientation were found in MDM vibrated using magnetic force than those vibrated using a shaking table. The orientation coefficient varied with the vibration method but had no obvious relationship to the fiber volume fraction. The orientation of steel fibers influenced the flexural strength of the specimen series with fiber volume fractions of 0.5% and 1.0%. The gain in strength is one of the material properties enhanced by fiber orientation. The benefits of fiber orientation on other material properties of concrete and mortar will be further investigated.

## Figures and Tables

**Table 1:** Mixture proportion of the concrete (by weight kg/m^3^).

Content	Water	Sand	Cement	River Stone/Steel Slag
Weight	480	144	730	1096

**Table 2:** Particle size distribution of coarse steel slag.

Size (mm)	<2.36	2.36	4.75	9.5	16	19	26.5	31.5
Retained (%)	99.8	96.3	94.0	86.8	59.2	29.0	3.6	0

**Table 3:** Splitting test results of test specimen series NC-S (normal concrete with shaking table vibration).

Specimen	7 Days		28 Days	
	*F* (kN)	*f*_ts_ (MPa)	*F* (kN)	*f*_ts_ (MPa)
NC-S1	42.9	2.34	47.8	2.61
NC-S2	41.9	2.29	46.9	2.56
NC-S3	42.3	2.31	47.1	2.57
NC-S4	42.9	2.34	48.4	2.64
NC-S5	41.6	2.27	46.7	2.55
NC-S6	43.1	2.35	47.4	2.59
Mean	42.5	2.32	47.4	2.59

**Table 4:** Splitting test results of test specimen series NC-M (normal concrete with magnetic orientation).

Specimen	7 Days		28 Days	
	*F* (kN)	*f*_ts_ (MPa)	*F* (kN)	*f*_ts_ (MPa)
NC-M1	41.2	2.25	45.6	2.49
NC-M2	40.5	2.21	45.2	2.47
NC-M3	41.4	2.26	46.0	2.51
NC-M4	41.5	2.27	44.7	2.44
NC-M5	40.1	2.19	45.4	2.48
NC-M6	41.0	2.24	45.6	2.49
Mean	40.9	2.24	45.4	2.48

**Table 5:** Splitting test results of test specimen series MDC-S (magnetically driven concrete-steel slag with shaking table vibration).

Specimen	7 Days		28 Days	
	*F* (kN)	*f*_ts_ (MPa)	*F* (kN)	*F* (kN)
MDC-S1	45.4	2.48	49.4	2.70
MDC-S2	45.2	2.47	50.1	2.74
MDC-S3	46.2	2.52	50.0	2.73
MDC-S4	45.1	2.46	49.6	2.71
MDC-S5	45.6	2.49	49.3	2.70
MDC-S6	45.2	2.47	50.4	2.75
Mean	45.5	2.48	49.8	2.72

**Table 6:** Splitting test results of the test specimen series MDC-M (magnetically driven concrete-steel slag with magnetic orientation).

Specimen	7 Days		28 Days	
	*F* (kN)	*f*_ts_ (MPa)	*F* (kN)	*f*_ts_ (MPa)
MDC-M1	44.1	2.41	47.1	2.57
MDC-M2	43.7	2.39	46.7	2.55
MDC-M3	43.6	2.38	47.3	2.58
MDC-M4	44.3	2.42	46.9	2.56
MDC-M5	44.5	2.43	47.2	2.57
MDC-M6	44.0	2.40	46.8	2.56
Mean	44.0	2.41	47.0	2.57

**Table 7:** Mix proportion of the magnetically driven mortar (by weight kg/m^3^).

Series	Water	Cement	Sand	Steel Slag	Iron Sand
A	178	310	764	1013	0
B	145	310	545	1013	0
C	138	310	434	1013	0
D	135	310	253	1013	0
E	193	310	382	1013	1670
F	185	310	556	1013	912

**Table 8:** Test specimens.

Test Specimen		*V*_f_ (%)	Vibration Method
Group A	S0-M1	0	Magnetic method
	S0-M2	0	Magnetic method
	S0-M3	0	Magnetic method
	S0.5-M1	0.5	Magnetic method
	S0.5-M2	0.5	Magnetic method
	S0.5-M3	0.5	Magnetic method
	S1-M1	1.0	Magnetic method
	S1-M2	1.0	Magnetic method
	S1-M3	1.0	Magnetic method
	S1.5-M1	1.5	Magnetic method
	S1.5-M2	1.5	Magnetic method
	S1.5-M3	1.5	Magnetic method
	S2-M1	2.0	Magnetic method
	S2-M2	2.0	Magnetic method
	S2-M3	2.0	Magnetic method
Group B	S0-S1	0	Shaking table
	S0-S2	0	Shaking table
	S0-S3	0	Shaking table
	S0.5-S1	0.5	Shaking table
	S0.5-S2	0.5	Shaking table
	S0.5-S3	0.5	Shaking table
	S1-S1	1.0	Shaking table
	S1-S2	1.0	Shaking table
	S1-S3	1.0	Shaking table
	S1.5-S1	1.5	Shaking table
	S1.5-S2	1.5	Shaking table
	S1.5-S3	1.5	Shaking table
	S2-S1	2.0	Shaking table
	S2-S2	2.0	Shaking table
	S2-S3	2.0	Shaking table

Note: *V*_f_ is fiber volume fraction.

**Table 9:** Test results of fiber orientation.

Group A					Group B				
**Specimen**	**0**–**15°**	**15°**–**45°**	**45°**–**75°**	**75°**–**90°**	**Specimen**	**0**–**15°**	**15°**–**45°**	**45°**–**75°**	**75°**–**90°**
S0.5-M1	177	58	31	15	S0.5-S1	68	80	53	56
S1.0-M1	307	73	48	34	S1.0-S1	85	88	99	80
S1.5-M1	368	84	64	42	S1.5-S1	117	138	122	100
S2.0-M1	468	96	80	54	S2.0-S1	165	188	133	142
